# Decoding Cold Therapy Mechanisms of Enhanced Bone Repair through Sensory Receptors and Molecular Pathways

**DOI:** 10.3390/biomedicines12092045

**Published:** 2024-09-09

**Authors:** Matthew Zakaria, Justin Matta, Yazan Honjol, Drew Schupbach, Fackson Mwale, Edward Harvey, Geraldine Merle

**Affiliations:** 1 Surgical and Interventional Sciences Division, Faculty of Medicine, McGill University, Montreal, QC H3A 2B2, Canada; matthew.zakaria@mail.mcgill.ca (M.Z.); justin.matta@mail.mcgill.ca (J.M.); yazan.honjol@mail.mcgill.ca (Y.H.); drew.schupbach@mail.mcgill.ca (D.S.); edward.harvey@mcgill.ca (E.H.); 2 Department of Surgery, Faculty of Medicine, McGill University, Montreal, QC H3A 0C5, Canada; 3 Lady Davis Institute for Medical Research, Lady Davies Institute Jewish General Hospital, 3755 Cote-St. Catherine Road, Room 602, Montréal, QC H3T 1E2, Canada; fackson.mwale@mcgill.ca; 4 Department of Chemical Engineering, École Polytechnique de Montréal, Montreal, QC H3T 1J4, Canada

**Keywords:** cold, bone healing, tissue engineering, hypoxia, vasculature, osteogenesis, shock proteins, osteoblasts

## Abstract

Applying cold to a bone injury can aid healing, though its mechanisms are complex. This study investigates how cold therapy impacts bone repair to optimize healing. Cold was applied to a rodent bone model, with the physiological responses analyzed. Vasoconstriction was mediated by an increase in the transient receptor protein channels (TRPs), transient receptor potential ankyrin 1 (TRPA1; *p* = 0.012), and transient receptor potential melastatin 8 (TRPM8; *p* < 0.001), within cortical defects, enhancing the sensory response and blood flow regulation. Cold exposure also elevated hypoxia (*p* < 0.01) and vascular endothelial growth factor expression (VEGF; *p* < 0.001), promoting angiogenesis, vital for bone regeneration. The increased expression of osteogenic proteins peroxisome proliferator-activated receptor gamma coactivator (PGC-1α; *p* = 0.039) and RNA-binding motif protein 3 (RBM3; *p* < 0.008) suggests that the reparative processes have been stimulated. Enhanced osteoblast differentiation and the presence of alkaline phosphatase (ALP) at day 5 (three-fold, *p* = 0.021) and 10 (two-fold, *p* < 0.001) were observed, along with increased osteocalcin (OCN) at day 10 (two-fold, *p* = 0.019), indicating the presence of mature osteoblasts capable of mineralization. These findings highlight cold therapy’s multifaceted effects on bone repair, offering insights for therapeutic strategies.

## 1. Introduction

Novel modalities in the approach to bone repair may address the burdens that patients face, especially for aging populations and in regard to complications concerning bone density [1]. One such avenue may be the usage of cold therapy, where intermittent short-term cold exposure has been shown to lead to increased bone healing dynamics [2,3]. However, a major bottleneck in the ideal application of cold exposure is a lack of understanding of the mechanisms of action. Numerous in vivo studies and observations have shown that prolonged cold exposure impedes bone growth, repair, and morphology [4,5,6], while short-term cold exposure exerts beneficial effects on bone healing and morphology [3,7,8,9]. In vitro investigations have demonstrated that long-term cold exposure inhibits specific pathways associated with bone healing, such as osteoblastogenesis and hypoxia signaling [4,5,10,11,12,13]. Conversely, short-term cold exposure induces the upregulation of these pathways, but such mechanisms have yet to be prominently identified within an in vivo bone injury model [14,15,16].

Several investigations into the short-term application of cold have elucidated its potential for augmenting bone development and repair, both in vitro and in vivo. In vivo studies have consistently demonstrated enhanced bone formation post-injury, characterized by the increased deposition of bone tissue at the injury site, concomitant with elevated levels of osteoblast activity biomarkers, such as alkaline phosphatase (ALP) [2,3,17]. Additionally, in vitro experiments have revealed that cold stimulation of mesenchymal stem cells (MSCs) and osteoblast progenitors promotes osteoblast differentiation [14].

Collectively, cold exposure initiates a shift in cellular expression, especially within bone cells, stimulating anabolic bone formation pathways. Established reports in the literature have shown that cold exposure induces rapid cellular responses, largely mediated by the activation of cold-shock proteins [18,19]. RNA-binding motif protein 3 (RBM3), in particular, has been established as the most representative protein expressed under cold shock [20]. Similarly, peroxisome proliferator-activated receptor gamma coactivator 1-alpha (PGC-1α) is a key regulator in bone homeostasis, affecting primarily osteoblastogenesis, as well as mitochondrial biogenesis [21]. Both cold-shock proteins have a prominent role in the cellular adaptation necessary for cellular survival [22,23]. Recently, these proteins have garnered particular attention for their potential impact on bone repair, due to their prevalence in osteoblastic cells and their pivotal role in regulating osteoblastogenesis pathways [19]. Additionally, it has been previously established that compromise of the vascular network in and around bone results in hypoxia, leading to the activation of a key mechanism in bone healing to restore blood flow, i.e., angiogenesis [24]. Angiogenesis is a critical part of the bone healing process, in order to restore native blood flow following a bone injury [25]. In vivo bone injury studies have shown enhancement of the vasculature following cold treatment, with angiogenic markers, such as the vascular endothelial growth factor (VEGF), being upregulated, which is indicative of the enhancement of angiogenic pathways. However, there is a lack of findings regarding the mechanism of action at the bone injury site [3,17]. Reports have shown that short-term cold exposure effects bone perfusion [26]; for instance, 10 min of immersion in water at 22 °C causes an approximately 40% reduction in whole-limb blood flow [7].

The TRP (transient receptor potential) family of receptors are expressed in sensory neurons and vascular endothelial cells. These receptors play a crucial role in sensing various stimuli, including temperature changes [27,28]. Among the TRP family, TRP channels, such as the transient receptor potential cation channel subfamily M (TRPM8) and transient receptor potential ankyrin 1 (TRPA1), are particularly involved in the perception of cold temperatures and in the conduction of this vasoconstrictive pathway [29]. TRPM8 channels are pivotal in coordinating the response to cold exposure, within a temperature range typically from 8 °C to 28 °C [30]. TRPA1 channels are activated by cold exposure within a range from approximately 8 °C to 17 °C, causing vasoconstriction and changes to blood flow, which are essential for thermoregulation and tissue viability during cold exposure [31].

**Figure 1:**
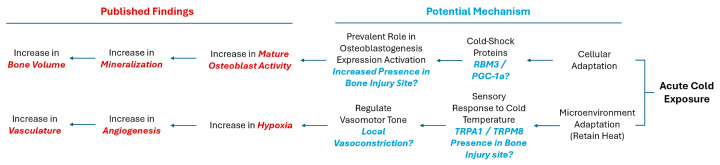
Potential mechanisms activated in response to cold exposure.

With this in mind, we hypothesized that with the application of cold, there may be an increase in the presence of RBM3 and PGC-1α within the bone injury site, which could facilitate the differentiation of osteoblasts and explain documented cases of increased mineralization of newly formed bone (Figure 1). Additionally, exposure to cold requires a microenvironmental adaptation to maintain heat homeostasis that involves a sensory response to the cold temperature, leading to the activation of TRPA1 and TRPM8 channels. This in turn may highlight localized vasoconstriction that explains the increase in hypoxia detected and, subsequentially, the enhanced vasculature network within bone injury sites (Figure 1).

The objective of this study is to delineate the mechanism by which cold therapy affects bone formation in vivo at the injury site. Building on the potential benefits of acute cold observed in our previous studies, we have extended our investigation to the mechanisms through which cold exposure promotes osteogenesis and contributes to the formation of a bony callus. In this work, we have implemented a simple and reproducible mouse defect model to demonstrate that the localized application of a cold temperature indirectly leads to the elevated induction of hypoxia in the bone injury site, by modifying the vasomotor tone through TRP-related pathways, in conjunction with the increased detection of RBM3 and PGC-1a, responsible for the upregulation of osteoblast differentiation (Figure 1).

## 2. Materials and Methods

### 2.1. Cortical Defect Animal Model

Following approval from the McGill Facility Animal Care Committee, in accordance with the Canada Council on Animal Care and NIH guidelines, a bilateral cortical bone defect model was applied to ten male C3H-strain mice aged 2–3 months old (Charles River Laboratories). Moreover, 30 min prior to the surgical intervention, each animal was given subcutaneous injections of an analgesic slow-release buprenorphine and prophylactic antibiotics, Baytril, for pain management and infection prevention, respectively. The mice were then sedated in a sedation box, with an influx of 4% isoflurane and pure oxygen at a flow rate of 1.0 LPM, and maintained under anesthesia, with an influx of 2.5–3.0% isoflurane at a flow rate of 0.5 LPM, provided through an airflow mask. To ensure thermal regulation, a heating pad was employed and hydration was maintained with the administration of a warm saline solution (0.2–0.5 mL/10 g, subcutaneously). A lateral 5 mm initial incision was made referencing the trochanter; the muscles were bluntly dissected longitudinally to gain access to the femur. Rectangular cortical defects (1 mm × 2.5 mm) were made within the ventrolateral aspect of each of the femoral diaphysis, using a 1 mm high-speed burr (Stryker, Hamilton, ON, Canada) drill (Figure S1). Following the formation of the defect, the region was flushed with phosphate buffered saline (PBS) to remove any remaining bone fragments.

### 2.2. Hypoxyprobe Treatments

Seven days after the operation on the mice, the Hypoxyprobe treatments commenced. Hypoxyprobe^TM^ (Hypoxyprobe Inc., Burlington, MA, USA) was diluted in saline to reach a final concentration of 2.45 mg/mL, in accordance with the manufacturer’s guidelines, and 1.5 mL of the solution was intraperitoneally injected (Figures S1 and S2). Five minutes after the injection took place, the experimental hindlimb of the mouse was exposed to an ice-water bath for fifteen minutes. Only the hindlimb of interest was immersed in the ice-water bath. An internal temperature of 19 degrees Celsius within the mouse hindleg can be obtained through following the established methodology [3]. The mice exhibited healthy behavior throughout the duration of the experiments. The cortical defects formed resulted in no inhibitions of the animal behavior. No issues arose concerning skin sensitivity during the PO treatment.

### 2.3. Tissue Harvesting and Staining Analysis

Immediately after the Hypoxyprobe treatments, the mice were then euthanized, firstly by sedating them with an influx of 5% isoflurane at a flow rate of 4.0 LPM in an induction chamber until they became unconscious, followed by an influx of pure carbon dioxide until there was no evidence of heart palpitations, after which the mice were left in the induction chamber for 2 more minutes. Lastly, in accordance with McGill FACC guidelines, chemical asphyxiation was followed by physical asphyxiation via cervical dislocation. The femurs were then harvested and fixated in 4% paraformaldehyde for 48 h. The femurs were then decalcified with 10% EDTA, paraffin embedded, and cut into 5 µm sections, using a LEICA 2255 microtome (Leica Microsystems, Concord, ON, Canada). Immunohistochemistry was conducted, utilizing a secondary antibody approach. Initially, irreversible protein adducts formed between pimonidazole and hypoxic cells (See Supplementary Information) were detected in the mice, following the Hypoxyprobe experiment design, using an anti-pimonidazole antibody (Hypoxyprobe Inc). Additionally, cold-shock proteins RBM3 and PGC-1a were assessed. The detection utilized a secondary antibody sequence, with a fluorescein isothiocyanate (FITC)-conjugated system. Sections were incubated overnight with primary antibodies, followed by goat anti-rabbit IgG, conjugated with HRP as a secondary antibody. Antibody binding was detected using the DAKO antigen retrieval system (DAB kit, K3468, USA, Agilent, Santa Clara, CA, USA). Sections were counterstained with Fast Green (Abcam, ab146267, Abcam Inc., Waltham, MA, USA), dehydrated, cleared, and mounted. Immunofluorescence staining was carried out for CD34 (Santa Cruz, sc-74499 FITC, 1:250, Dallas, TX, USA) in endothelial vascular cells, vascular endothelial growth factor (VEGF, Santa Cruz, sc-7269 FITC, 1:250) as an angiogenic factor, transient receptor potential cation channel subfamily A member 1 (TRPA1, Santa Cruz, sc-376495, 1:250), and transient receptor potential melastatin 8 (TRPM8, Novus Biotech, NB200-145F, 1:250, Centennial, CO, USA), all involving overnight incubation with primary antibodies and subsequent analysis.

Microscopic images of the immunohistochemical stained samples were captured using either (1) a Zeiss Axioskop 40 microscope (Carl Zeiss, Toronto, ON, Canada) or (2) an Aperio digital pathology slide scanner from Leica Biosystems. Images of the immunofluorescent-stained samples were captured using an EVOS M5000 Imaging System (Life Technologies Corporation, Bothell, WA, USA). Histomorphometric calculations of the stained cell percentages, in defined regions of interest, were performed using ImageJ v.1.6.0 software (NIH, Bethesda, MD, USA). SPSS version 20 (IBM) was used to compare the means of the parameters in the control and experimental groups, using a paired Student *t*-test, with statistical significance set at *p* < 0.05.

### 2.4. Femoral Fracture Animal Model

The next segment of this study was carried out following approval from the McGill Facility Animal Care Committee and in adherence with the guidelines from the Canada Council on Animal Care and National Institutes of Health. Bilateral femoral fractures were induced in 21 male C3H-strain mice, selected for their distinct bone characteristics. Moreover, 30 min prior to the surgical intervention, each animal was given subcutaneous injections of an analgesic slow-release buprenorphine and prophylactic antibiotics, Baytril, for pain management and infection prevention, respectively. The mice were then sedated in a sedation box, with an influx of 4% isoflurane and pure oxygen at a flow rate of 1.0 LPM, and maintained under anesthesia, with an influx of 2.5–3.0% isoflurane at a flow rate of 0.5 LPM, provided through an airflow mask. To ensure thermal regulation, a heating pad was employed and hydration was maintained with the administration of a warm saline solution (0.2–0.5 mL/10 g, subcutaneously).

### 2.5. Treatments and Cell Study

The same cold treatment was applied daily for five days in 9 mice, 10 days in 9 mice, and 14 days in 3 mice. A summary of the study segments has been provided in Supplementary Figure S3. The mice were then euthanized, firstly by sedating them with an influx of 5% isoflurane at a flow rate of 4.0 LPM in an induction chamber until they became unconscious, followed by an influx of pure carbon dioxide until there was no evidence of heart palpitations, after which the mice were left in the induction chamber for 2 more minutes. Lastly, in accordance with McGill FACC guidelines, chemical asphyxiation was followed by physical asphyxiation via cervical dislocation. To prevent contamination, the whole animals were transported to a sterile cell culture hood in 70% ethanol solution. The femurs were then isolated and a puncture needle was used to enter the medullary cavity of the bone to aspirate the bone marrow. The femurs were then washed 3 times with PBS. To maximize the amount of osteoblast extraction, each group consisted of 3 femurs paired together and crushed coarsely inside an MEM (Gibco 12561056, Thermo Fisher Scientific, Waltham, MA, USA) enriched with amino acids (Gibco 50X MEM Amino Acids, 11130051 Thermo Fisher Scientific), fetal bovine serum (12483020, Thermo Fisher Scientific), and penicillin–streptomycin (15140122, Thermo Fisher Scientific). A digestion sequence followed, in order to specifically isolate osteoblast cells using a modification of pre-existing procedures from the literature [32]. Bone fragments were digested sequentially in 10 mL of each of the following enzymes: TrypL (1 mg/mL; 12605010 Thermo Fisher Scientific), dispase II (2 mg/mL; 17105041 Thermo Fisher Scientific), and twice in collagenase II (3 mg/mL; 17101015 Thermo Fisher Scientific). The supernatants from the trypsin and dispase digestions were discarded, whereas those from the collagenase digestions were retained and combined. The cells were then assessed in terms of their morphology to confirm them as osteoblasts. The OBs were then cultured until confluency was reached. Once confluency was attained, Alamar blue cell viability testing was conducted to normalize the cell counts.

The secretion level of the biomarkers related to osteoblast differentiation was measured after adding TrypL for the dissolution of OBs. The 2mL cell suspensions were then transferred into Eppendorf tubes and underwent lysing. The supernatant was carefully collected and, first, underwent an assessment of the osteocalcin (OCN) via an ELISA (ab285236, Abcam), followed by alkaline phosphatase (ALP) assessment via a colorimetric assay (ab83369, Abcam). Statistical analyses of the assays were conducted by taking absorbance measurements using a Varioskan LUX microplate reader (Thermo Fisher Scientific, N16044), with 406 nm absorbance for the ALP readings and 450 nm absorbance for the OCN readings. Statistical analysis was conducted using SPSS version 20 (IBM) via an unpaired Student *t*-test, with significance set at *p* < 0.05.

Another group of 3 mice, using the femoral fracture model, were used to test for mineralization, following daily cold exposure. The bilateral femoral fracture model mentioned was used, followed by daily usage of the same cold treatment regime for 14 days. Following osteoblast isolation after day 14, the cells were cultured in a 6 well plate and stained using alizarin red, to assess for mineralization nodules found within the osteoblasts. Histomorphometric calculations of the stained-cell percentages, in defined regions of interest, were performed using ImageJ v.1.6.0 software (NIH, Bethesda, MD, USA). SPSS version 20 (IBM) was used to compare the means of the parameters in the control and experimental groups, using a paired Student *t*-test, with statistical significance set at *p* < 0.05.

### 2.6. Statistical Design and Analysis

The purpose of this study is to assess the mechanisms upon which cold therapy positively affects bone healing, through a comparison of 2 means representing the presence of specific biomarkers affiliated with the potential repair pathways. Statistical power analysis was performed for sample size estimation (clinicalc.com/stats/samplesize) and an estimated sample size was calculated that was required for a significance level of 0.05 and power of 0.9. We used our previous pilot study for the anticipated means and a standard deviation of +/−5.5 [3]. All the assessments were performed in a blinded manner regarding the source of the tissue. To prevent asymmetric expression in the osteoblast differentiation/mineralization, right or left limbs were used as an experimental group. Student paired *t*-tests were used to assess the quantitative data for paired femora from the same mouse to account for varying baseline fluctuation between the mice, while unpaired *t*-tests were used to account for two groups of means unaffected by baseline fluctuations between the mice. Statistical analysis was conducted using SPSS version 20 (IBM). Differences were considered significant at *p* < 0.05.

## 3. Results

**Figure 2:**
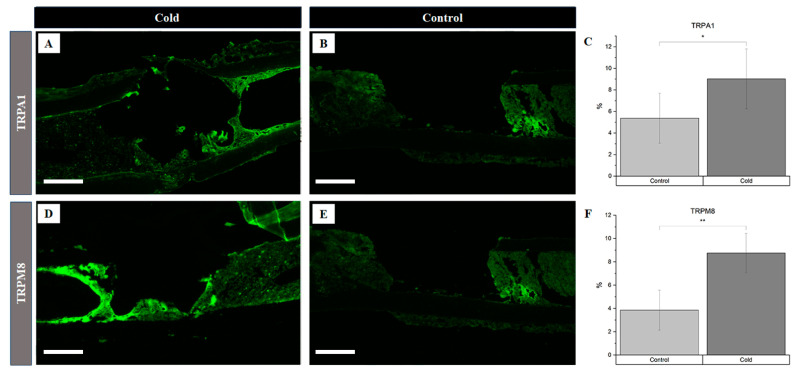
Identification of receptor proteins involved in vasoconstriction within a cortical defect following cold exposure. TRPM8 and TRPA1 detection (bright green) within the cortical defect region (4× magnification). Scale bar represents 500 µm. (**A**,**D**) are non-treated femurs serving as a baseline. (**B**,**E**) are cold-treated femurs. (**C**) TRPA1 staining expression analysis (*n* = 8) of positive staining for TRPA1 in the control group was 5.38 ± 2.32, while in the experimental group it was 9.03% ± 2.78 (* *p*-value = 0.012). (**F**) TRPM8 staining expression analysis (*n* = 8) of positive staining for TRPM8 in the control group was 3.86% ± 1.72, while in the experimental group it was 8.75% ± 1.68 (** *p*-value < 0.001).

The assessment of the vasoconstriction receptors revealed a significant increase (Figure 2C) in TRPA1 presence following cold treatment (*p* = 0.012), with levels rising to 9.03% ± 2.78 in the cortical defect region, compared to 5.38 ± 2.32 in the untreated controls (Figure 2A vs. Figure 2B). Similarly, the TRPM8 levels were significantly elevated (*p* < 0.001) after cold exposure, showing an increase to 8.75 ± 1.68 in the treated femurs versus 3.86 ± 1.72 the untreated ones (Figure 2D vs. Figure 2E). An elevation in the receptors responsible for triggering the vasoconstrictive response confirms the detection of cold exposure within the microenvironment of the bone injury site.

**Figure 3:**
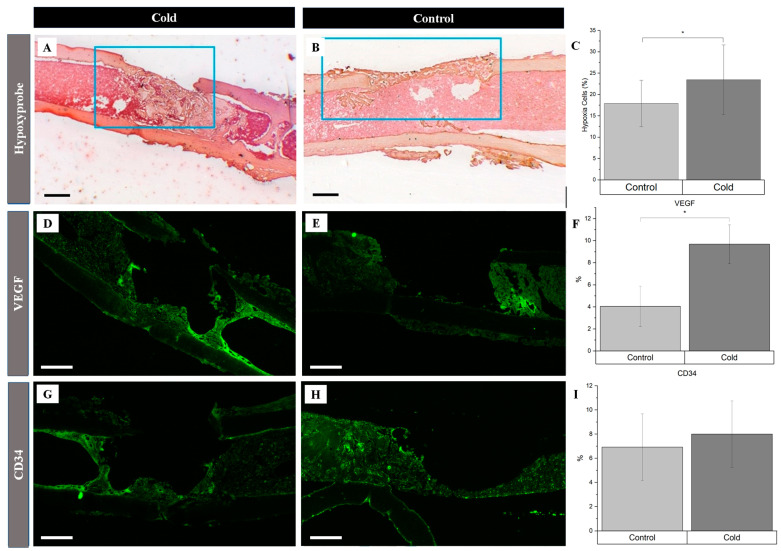
Identification of angiogenic-related factors and endothelial cells within a cortical defect following cold exposure. VEGF and CD34 (bright green) detection within the cortical defect region (4× magnification). Scale bar represents 500 µm. (**A**,**D**,**G**) are the cold-treated femurs. (**B**,**E**,**H**) are the non-treated femurs serving as a baseline. (**C**) Hypoxia staining expression analysis (*n* = 10) of positive staining for hypoxia in the control group was 17.9% ± 3.8, while in the experimental group it was 23.5% ± 05.8 (* *p*-value < 0.01). (**F**) VEGF staining expression analysis (*n* = 8) of positive staining for VEGF in the control group was 4.05% ± 1.83, while in the experimental group it was 9.69% ± 1.75 (* *p*-value < 0.001). (**I**) CD34 staining expression analysis (*n* = 8) of positive staining for CD34 in the control group was 6.92% ± 2.77, while in the experimental group it was 8.00% ± 2.76 (*p*-value = 0.42).

There was a 5.6% increase in the number of hypoxic cells within and around the cortical bone defect in the experimental (Figure 3A) hindlegs (23.5% ± 5.8) compared to the untreated (Figure 3B) controls (17.9% ± 3.8) following exposure to an acute cold stimulus (*p*-value < 0.01), reflecting an increase in local hypoxia following cold exposure (Figure 3C). Investigating the cold-induced cortical defect repair revealed significant angiogenic changes, particularly for vascular endothelial growth factor (VEGF) expression. VEGF expression in the cold-treated group (Figure 3D) notably surpassed the control group (Figure 3E), with levels of 9.69% ± 1.75 and 4.05% ± 1.83, respectively (Figure 3F; *p* < 0.001), suggesting accelerated angiogenesis. Endothelial cells within the cortical defect were identified using the endothelial-specific marker CD34. There was no statistically significant change (Figure 3I) in the presence of these cells in the cold-treated group (8.00% ± 2.76; Figure 3G) compared to the control group (6.92% ± 2.77; Figure 3H).

**Figure 4:**
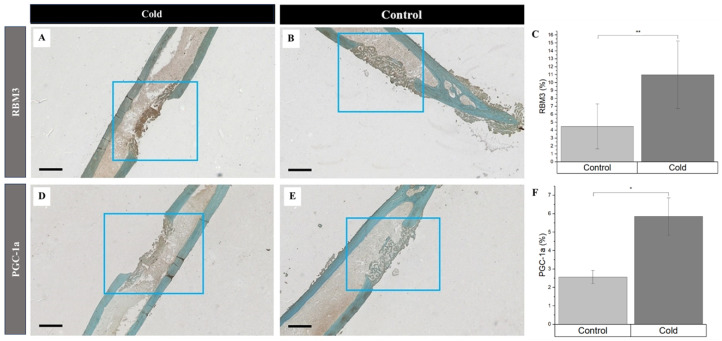
Identification of cold-shock proteins and hypoxia in regenerating bone following cold exposure. PGC-1a (brown), RBM3 (brown), and hypoxia (brown) detection within the cortical defect region (4x magnification). Scale bar represents 500 µm. (**A**,**D**) are cold-treated femurs. (**B**,**E**) are non-treated femurs serving as a baseline. (**C**) RBM3 staining expression analysis (*n* = 8) of positive staining for RBM3 in the control group was 4.47% ± 2.84, while in the experimental group it was 11.0% ± 4.26 (** *p*-value < 0.008). (**F**) PGC-1a staining expression analysis (*n* = 8) of positive staining for PGC-1a in the control group was 2.57% ± 0.36, while in the experimental group it was 5.85% ± 1.01 (* *p*-value = 0.039).

Simultaneously, the increased detection of PGC-1a and RBM3 were prevalent in newly formed bone cells and the surrounding matrix within the cortical defects following cold exposure. RBM3 significantly (*p*-value < 0.008) increased (Figure 4C) within cold-treated femora (Figure 4A) (11.0% ± 4.26%) compared to non-treated (Figure 4B) femora (4.47% ± 2.84%)). Synonymously, PGC-1a was significantly (*p*-value = 0.039) elevated (Figure 4F) in cold-treated cortical defects (Figure 4D) (5.85% ± 1.01%) in comparison to non-treated (Figure 4E) defects (2.57% ± 0.36%). An increase in the presence of RBM3 and PGC-1a indicates that cells within the bone injury site are affected by cold exposure, resulting in the stimulation of proteins responsible for cellular adaptation required for cell survival [19,33,34]. Furthermore, the percentage of PGC-1a and RBM3 positive cells marked by DAB staining (brown) within and around the cortical bone defect in the hindlimbs of mice exposed to a cold stimulus was higher in comparison to the control hindlimbs, which is of particular interest, in part, due to the prominent roles RBM3 and PGC-1a have in osteoblastogenesis pathways.

**Figure 5:**
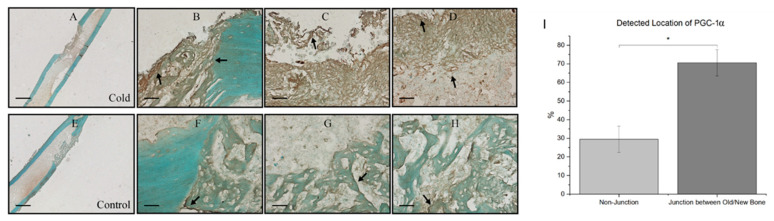
Prevalence of PGC-1a at the junction of old and new bone formation following cold therapy treatment. (**A**,**E**) at 2.5× magnification. Scale bar represents 500 µm. (**B**–**D**,**F**–**H**) at 20× magnification. Scale bar represents 50 µm. PGC-1a has been shown to be upregulated (**I**) at the junction of new and old bone. (**F**–**H**) Furthermore, PGC-1a expression within these junctions appears to be elevated following cold exposure (**B**–**D**) indicating PGC-1a upregulation. (**I**) Detected location of PGC-1α staining expression analysis (*n* = 8) of PGC-1α within cells at the junction of newly formed bone and old bone was 70.56% ± 7.08, while in non-junctional regions it was 29.44% ± 7.08 (* *p*-value < 0.00001).

At the junction of old bone and new bone formation, there was a distinct observational contrast in the prevalence of PGC-1a within the new bone formation region within cold-treated femora (Figure 5A) and non-treated femora (Figure 5E). Within both the cold-treated and control femora, 70.56% ± 7.08 of total PGC-1a was detected at the junction of new and old bone (Figure 5B–D) corresponding with PGC-1a’s role as a cold-shock protein and its role in deterring necessary cellular changes, as well as PGC-1a’s prominence in the bone repair process, as this finding was also consistently found within non-treated legs (Figure 5F–H).

**Figure 6:**
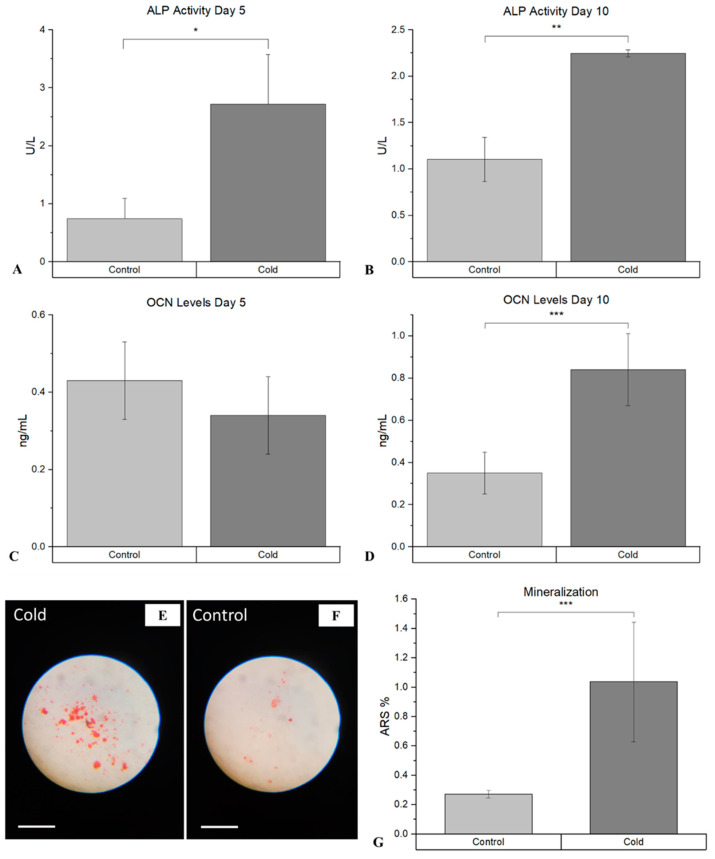
Detection of ALP and OCN at day 5 and day 10 within isolated osteoblasts following cold exposure and mineralization within isolate osteoblasts following cold exposure for 14 days. (**A**) ALP activity after 5 days of cold exposure was 2.72 U/L (µmol/min/L) ± 0.85, while in the non-treated group it was 0.74 U/L ± 0.35 (* *p*-value = 0.021). (**B**) ALP activity after 10 days of cold exposure was 2.25 U/L ± 0.04, while in the non-treated group it was 1.0301 U/L ± 0.24 (** *p*-value < 0.001). (**C**) OCN levels after 5 days of cold exposure was 0.34 mg/mL ± 0.10, while in the non-treated group it was 0.43 mg/mL ± 0.10 (*p*-value = 0.29). (**D**) OCN levels after 10 days of cold exposure was 0.84 mg/mL ± 0.17, while in the non-treated group it was 0.35 mg/mL ± 0.10 (*** *p*-value = 0.019). ARS (red) detection within isolated osteoblasts (10× magnification). Scale bar represents 250 µm. (**E**) Osteoblasts isolated from non-treated fractured femurs. (**F**) Osteoblasts isolated from daily cold-treated fractured femurs. (**G**) ARS staining analysis: Detection of ARS after 14 days of cold exposure was 1.04% ± 0.40, while in the non-treated group it was 0.27% ± 0.025 (*** *p*-value = 0.030).

The assessment of the differentiation of osteoblasts following cold therapy shows that there was a statistically significant facilitative impact on the osteogenic pathway. ALP, an early marker of immature osteoblasts, was shown to be significantly elevated (*p*-value = 0.021) following cold treatment at day 5 (Figure 6A), compared to the non-treated femurs (2.72 U/L ± 0.85 vs. 0.74 U/L (µmol/min/L) ± 0.35). This similar trend was prevalent at day 10 (Figure 6B), as well where ALP was significantly elevated (*p*-value < 0.001) following cold exposure (2.25 U/L ± 0.04 vs. 1.03 U/L ± 0.74). OCN, a marker for mature osteoblasts capable of mineralization, was not significant at day 5 (*p*-value = 0.29; Figure 6C).

However, OCN was significantly (*p*-value = 0.019) more prominent within cold-treated femurs (0.84 mg/mL ± 0.17 vs. 0.35mg/mL ± 0.10) at day 10 (Figure 6D), indicating the presence of mature osteoblasts capable of mineralization. ARS further confirmed the detection on calcium deposits, with a statistically significant increase (*p*-value = 0.030) in the amount of mineralization from isolated osteoblasts at day 14 (Figure 6G) following cold exposure (Figure 6E), in comparison to the non-treated (Figure 6F) samples (1.04% ± 0.40 vs. 0.27% ± 0.025).

## 4. Discussion

The biological mechanism that promotes reparative processes and osteogenic differentiation upon cold exposure is multifaceted, as different pathways related to bone repair are upregulated. Short-term cold exposure elicits a complex physiological response involving vasoconstriction, impacting bone healing through the modulation of angiogenesis and vascular responses [7,27]. The hypoxic microenvironment ensuing from diminished blood flow elicits the production of angiogenic factors, such as the vascular endothelial growth factor (VEGF) [35]. Adaptations to cold exposure lead to the activation of cell hemostasis regulators within bone cells that corroborate the stimulation of osteogenic pathways and bone formation.

**Figure 7:**
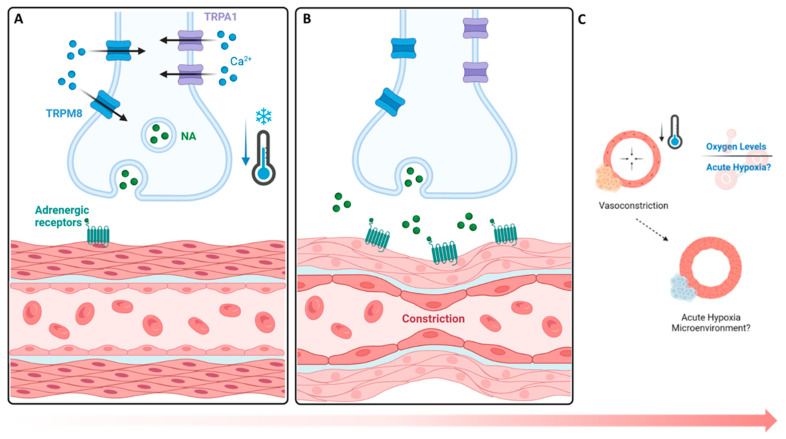
Cold-based activation of vasoconstrictive pathways and hypoxic impact. (**A**,**B**) TRPA1 and TRPM8 cold exposure mechanism leads to the activation of TRPM8 (8–28 °C) and TRPA1 (8–17 °C), initiating a contractive response leading to vasoconstriction. (**C**) Hypoxic impacts. A reduction in the influx of blood supply coincides with a decrease in oxygen availability, leading to the formation of an acute hypoxic microenvironment with respect to nearby cells. NA: norepinephrine.

The findings of this study show that an upregulation of TRPA1 (3.65% increase, Figure 2) and TRPM8 (4.89% increase, Figure 2) occurs, followed by a hypoxic environment (5.0% increase, Figure 3), upon cold application. Given their role in sensory perception, cold-stimulated TRPA1 and TRPM8 upregulation within cortical defects signifies an amplified sensory response to a lower temperature [27]. The vasoconstriction, a pivotal process in blood flow regulation, is induced by the sympathetic nervous system through the activation of TRPA1 and TRPM8, which release norepinephrine, activating α-adrenergic receptors in vascular smooth muscle cells (Figure 7A) [31,36]. Then, an intracellular signaling cascade occurs with the release of intracellular calcium ions, which causes the contraction of vascular smooth muscle cells (Figure 7B), ultimately resulting in vasoconstriction and reduced blood flow to peripheral tissue, leading to hypoxia (Figure 7C) [27]. To measure the acute hypoxic microenvironment, we developed a novel approach based on pimonidazole-based staining [37] to measure the hypoxic cells within a bone injury site (Figures S1A and S4), as the short half-life of the hypoxia inducible factor 1-α protein is impractical for directly analyzing the level of hypoxia [10].

**Figure 8:**
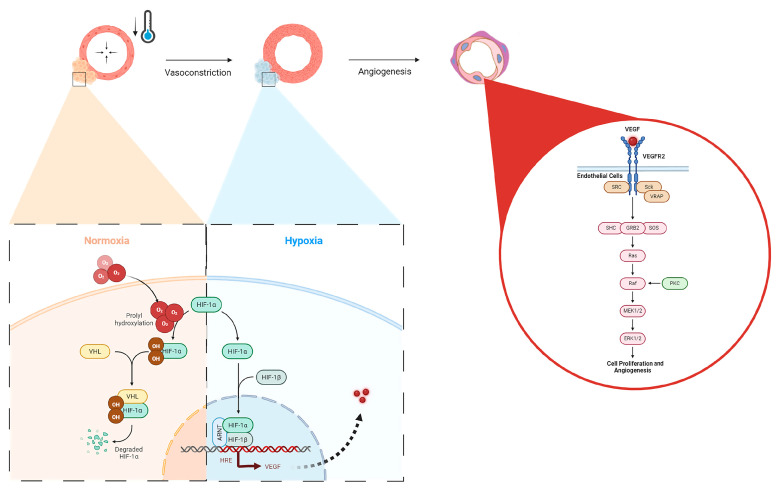
Hypoxic upregulation following vasoconstriction via local cold exposure and the impact on angiogenic pathways. Under normal oxygen conditions, HIF-1a is degraded via von Hippel–Lindau (VHL) disease after prolyl hydroxylation, but in hypoxia, HIF-1a forms a dimer with HIF-1B, nucleolocalizes with the aryl hydrocarbon receptor nuclear translocator (ARNT), leading to VEGF expression through the HRE pathway, which then activates VEGFR2 in endothelial cells, upregulating cellular proliferation and angiogenesis via the Ras/Raf signaling pathway.

In the context of bone healing, angiogenesis is essential for supplying oxygen, nutrients, and growth factors to the healing site, facilitating the formation of new bone tissue [11]. Here, we have shown that the application of localized, non-invasive cold therapy for a short duration of time has demonstrated an elevation in the baseline hypoxia within a bone injury site (23.5%, Figure 3) and an upregulation of VEGF (9.69%, Figure 3) in the cortical defect following cold therapy. Under normal conditions, the hypoxia inducible factor 1-alpha (HIF-1α) protein undergoes degradation because the presence of oxygen induces prolyl hydroxylation, marking it for targeted destruction but, in a localized, acute hypoxic microenvironment, HIF-1α is stabilized and forms a dimer with HIF-1β to activate the expression of VEGF through the hypoxia-response element (HRE) pathway (Figure 8). The resulting VEGF is released to trigger: (1) endothelial cell activation through its receptor vascular endothelial growth factor receptor 2 (VEGFR2), initiating signaling pathways, such as PI3K-Akt and MAPK, which are crucial for endothelial cell proliferation and migration (Figure 8) [38,39]; and (2) TRPA1 activation, which directs the development of the direction of vessel elongation [40]. Furthermore, elevated VEGF following short-term cold exposure has been shown to be consistent with the enhanced maturation of the vasculature within bone injury models [2,3,17], especially within calluses where the heightened presence of VEGF overlaps in the same regions with an elevated presence of CD34, a biomarker of mature endothelial cells essential for the vasculature [2]. Within cortical defects specifically, previous reports have shown that the bone vessel volume over the tissue volume (BVV/TV) was found to be higher in the cold-treated group compared with the control (7.8% vs. 6.7% *p* < 0.001) [3].

Nonetheless, TRPM8’s role in angiogenesis is less defined, but appears to be implicated in influencing the endothelial function, suggesting its potential contribution to the angiogenic process [40,41]. Consequently, the observed upregulation of VEGF suggests that an augmented angiogenic response occurs, essential for fostering bone regeneration within cortical defects [3,17]. Moreover, the collaborative action of VEGF, TRPA1, and TRPM8, indirectly supports osteogenic processes by securing an adequate blood supply to regenerating tissue, facilitating osteoblast proliferation and, thereby, promoting bone formation and maturation within cortical defects.

**Figure 9:**
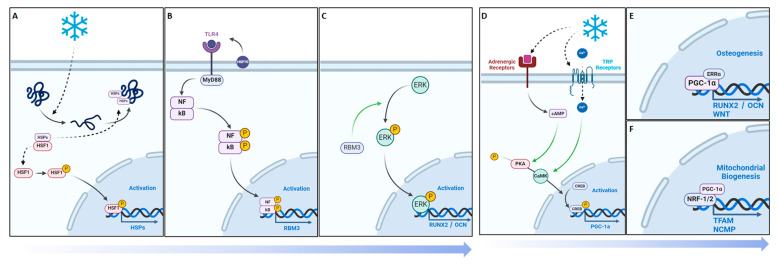
Cellular adaptive signaling pathways activated by cold exposure. RBM3 activation and downstream osteogenic potential following short-term cold exposure. (**A**) Cold exposure triggers a cellular cold-shock response, activating RBM3 via HSPs released by phosphorylated and nuclear-localized HSF1. (**B**) HSP70 activates TLR4, initiating the MyD88/NF-kB pathway, with phosphorylated NF-kB essential for RBM3 expression. (**C**) RBM3 activates the MAPK/ERK pathway, leading to Runx2 and osteocalcin (OCN) expression, crucial for osteoblast differentiation. PGC-1a activation and downstream osteogenic potential following short-term cold exposure. PGC-1α activation and osteogenic potential following short-term cold exposure. (**D**) Cold activation of adrenergic receptors and TRPs stimulates cAMP and calcium influx, leading to CREB phosphorylation via the PKA/Ca^2+^/CaMK pathway. (**E**) PGC-1α forms a complex with ERRα in osteoblastic cells, promoting differentiation via the Runx2/OCN pathway for mineralization. (**F**) PGC-1α and NRF-1/NRF-2 activate TFAM and NCMP, crucial for mitochondrial biogenesis and intracellular calcium storage.

While the vasomotor tone and hypoxia play an important role in regulating angiogenesis in bone repair following cold exposure, the expression of essential cell hemostasis regulators, such as RBM3 and PGC-1α, are elevated [42,43]. Initially, the cold denaturation of proteins results in the proliferation of heat-shock proteins (HSPs) for stabilization and proper folding. The dissociation of the HSP/heat-shock factor-1 (HSF1) dimer releases free HSF1, which becomes phosphorylated and nuclear localized, leading to HSP expression (Figure 9A). Heat-shock protein 70 (HSP70) activates Toll-like receptor 4 (TLR4), which triggers the MyD88/NF-kB signaling pathway. The phosphorylated and nuclear-localized NF-kB dimer is crucial for RBM3 expression (Figure 9B) [44,45]; RBM3 activates the MAPK/ERK pathway, leading to the expression of Runx2 and osteocalcin (OCN), essential for osteoblast differentiation and consistent with the preceding findings, showing that RBM3 has previously been shown to stimulate osteoblast differentiation via the ERK signalling pathway and activates two osteogenic genes, Runx2 and OCN (Figure 9C) [14,42]. Runx2 has been shown to be a critical gene in inducing osteoblast differentiation [46], whereas OCN has an important role in calcifying new bone tissue to reform the mechanical integrity of native bone at an injury site [47]. Several in vitro studies have shown the elevated expression of Runx2 following cold exposure stimulation, indicating that Runx2 is a crucial gene in the osteogenic pathway impacted by cold exposure [14,48]. Within fractures, this is important in transitioning from a fibrogenesis callus to a bony callus [49]. The increased expression of RBM3 confirms the activation of osteoblast differentiation associated with the elevation of ALP levels at days 5 and 10 (2.72 U/L, 2.25 U/L, Figure S4) and calcium-binding pathways, which are aligned with enhanced bone formation following acute cold therapy. This is consistent with in vitro studies that have shown elevated levels of ALP and OCN following short-term cold exposure, indicating the activation of osteoblastogenesis pathways essential for osteoblast maturation [14]. Under the same cold treatment conditions, ALP levels have been shown to increase in the callus of femoral fractures, correlating with enhanced mineralized bone formation. These results are consistent with similar observations in cortical defects, further supporting the role of cold exposure in stimulating bone repair pathways [2,3,17].

Studies have already demonstrated that cold exposure leads to the activation of PGC-1α via the PKA/CaMKII pathway, where cold activation of adrenergic receptors and transient receptor proteins (TRPs) stimulates cAMP and calcium influx, leading to cAMP-response element-binding protein (CREB) phosphorylation via the PKA/Ca^2+^/CaMK pathway (Figure 9D) [50,51]. PGC-1α has synergistic properties with ERRα, which is highly expressed in osteoblasts and osteoclasts, and interacts cooperatively with PGC-1α to amplify the levels of key proteins in bone formation, including OCN, Runx2, and osteopontin (OPN), necessary for mineralization (Figure 9E) [52]. For example, OPN facilitates blood vessel maturation following angiogenesis and is essential for early callus formation. [11] Furthermore, in vitro studies have demonstrated the upregulation of OPN, essential for osteoblast differentiation, following cold exposure, resulting from Runx2 stimulation [48]. The distinctive contrast between the PGC-1α detected at the junction between the formation of old bone and new bone further reinforces its importance in being responsible for establishing the baseline for new bone formed by osteoblasts to interconnect with old bone [53] (Figure 5). These osteoblasts then secrete further OPN and create a positive feedback cycle for bone regeneration [53]. Additionally, PGC-1alpha activation stimulates the transcription of mitochondrial genes by forming a complex with nuclear respiratory factor 1 and 2 (NRF-1 and NRF-2), activating mitochondrial transcription factor A (TFAM) and NCMP expression [50], which are crucial for mitochondrial biogenesis and intracellular calcium storage, enhancing oxidative phosphorylation and energy production (Figure 9F) [19]. This also induces the genes involved in calcium homeostasis, leading to increased calcium storage in mitochondrial granules, which is crucial for osteoblasts and bone formation [54]. Adequate calcium levels are essential for the mineralization process in osteoblasts. This is aligned with the elevated alizarin red staining in osteoblasts from cold-treated bone injury samples at day 14 (1.04%, Figure S5), indicating an enhanced extracellular matrix mineralization, reflecting improved osteoblast functionality and bone formation. This may be due to cold exposure influencing cellular metabolism and energy homeostasis via RBM3 and PGC-1α, supporting osteoblast differentiation and matrix mineralization (Figure S4). By boosting mitochondrial function and calcium storage, PGC-1alpha activation via cold therapy supports osteoblastic activity and bone formation. This dual action improves the mitochondrial efficiency and ensures sufficient calcium availability, facilitating proper osteoblast function and differentiation [54]. Future studies should further explore the influence of mitochondrial biogenesis on bone healing and matrix mineralization.

**Figure 10:**
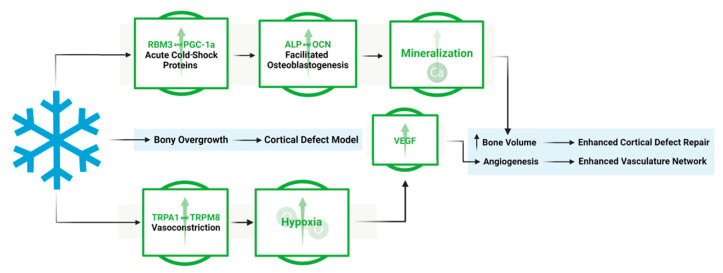
Overview of the mechanistic propensities of short-duration cold exposure and the potential impacts on a bone injury site. Blue: Observed impact of cold exposure on bone and vasculature morphology. Green: Observed impact of cold exposure on pathways involved in bone repair.

This work shows the dual effect of cold exposure on: (1) bone and vasculature morphology and (2) pathways involved in bone repair (Figure 10). The upregulation of TRPA1 and TRPM8 in the injury region induces vasoconstriction, resulting in a heightened acute hypoxic microenvironment, leading to elevated VEGF levels and the activation of angiogenic pathways, promoting a robust vasculature network in cold-treated bone injuries. Elevated RBM3 and PGC-1α levels at the injury site support osteoblast differentiation, necessary for new bone mineralization, thereby enhancing bone production following cold exposure.

## 5. Conclusions

In conclusion, the accelerated osteoblast differentiation, with increased ALP activity, and enhanced OCN-mediated bone matrix synthesis and mineral deposition, underscore the critical role of cold exposure in promoting advanced osteoblastic maturation and improving bone healing. The heightened presence of TRPA1 and TRPM8 within cortical bone defects highlights their pivotal involvement in cold-induced skeletal repair mechanisms. These transient receptor potential channels initiate vasoconstriction and trigger reparative responses crucial for bone regeneration, intensified by the observed increase in hypoxia following cold therapy. Elevated VEGF levels further support this process by stimulating angiogenesis, essential for fostering bone growth within cold-treated defects. The upregulation of RBM3 and PGC-1α underscores the adaptation of bone cells to cold stimulation, enhancing osteogenic processes and facilitating osteoblast differentiation, crucial for bone repair and regeneration. This comprehensive interplay between sensory perception, vascular regulation, angiogenesis, and osteogenesis, reveals the intricate dynamics behind cold-based therapeutic interventions for bone injuries, promising new avenues for future research and clinical applications. With the increase in interest in exosomes and further discoveries regarding the Jumonji C domain-containing (JMJD) protein family within the field of epigenetics regarding cold exposure and bone repair pathways [55,56,57,58], exosomes could be utilized to prime a bone injury microenvironment to respond favorably to external stimuli, such as cold, by optimizing bone repair pathways through miRNA and LncRNA-based epigenetic modification, locally. Pioneering research in biomaterials has begun to advance the development of 4D-based materials that respond to external stimuli, such as temperature, which formulate an active scaffold that can dynamically change over time allowing for an optimizable approach to bone healing [59,60,61,62,63].

## Data Availability

Dataset available on request from the authors.

## Supplementary Materials

The following supporting information can be downloaded at: https://www.mdpi.com/article/10.3390/biomedicines12092045/s1, Figure S1: Formation of the cortical bone defect within mouse femurs and the methodology of the Hypoxyprobe injections, Figure S2: Progression in the rationale for pursuing hypoxia. (A) Previous studies have shown that cold exposure leads to an increase in VEGF, which affects vascularity. This guided us to consider blood flow mechanics and how a decrease in blood flow, contrary to common perceptions in that an increase in blood flow is preferred for bone healing, can facilitate bone repair by potentially inducing an acute hypoxic microenvironment due to vasoconstriction. (B) Pimonidazole tags hypoxic cells through irreversible adduct formation that can then be detected via staining to provide relative hypoxic measurements on the impact of cold therapy on the microenvironment of the cortical defect, Figure S3: Schematic of the study design, Figure S4: Hypoxyprobe mechanism. Pimonidazole-based detection of hypoxia relies on the formation of irreversible adducts in acute hypoxic environments, Figure S5: Differentiation of osteoblasts and biomarkers present. Ref. [64] is mentioned in Supplementary File.
