# Supplementary material for: Decoding Cold Therapy Mechanisms of Enhanced Bone Repair through Sensory Receptors and Molecular Pathways
Source: Biomedicines. 2024 Sep 9;12(9):2045. doi: 10.3390/biomedicines12092045 (PMC11429201; doi:10.3390/biomedicines12092045)
Supplement: Supplementary file 1 [file biomedicines-12-02045-s001.zip › biomedicines-3163433-supplementary.pdf]

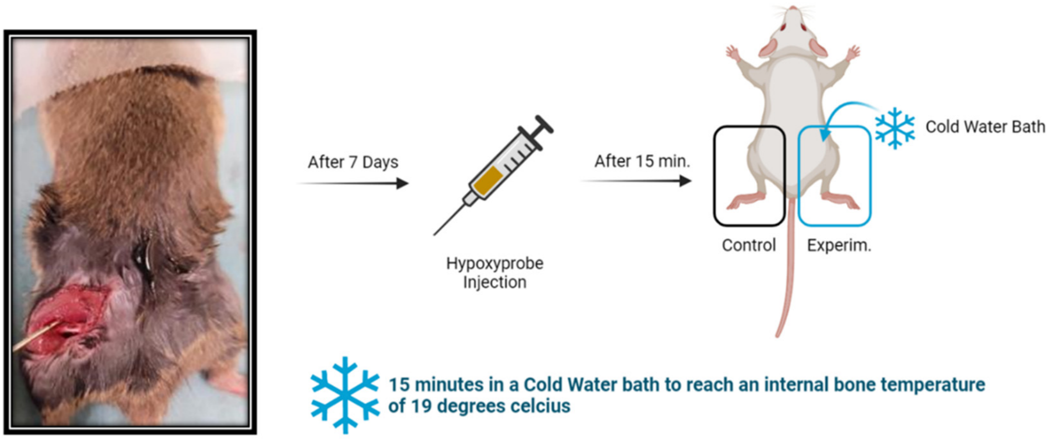

Figure S1. Formation of the cortical bone defect within the mouse femurs and the methodology of the hypoxyprobe injections.

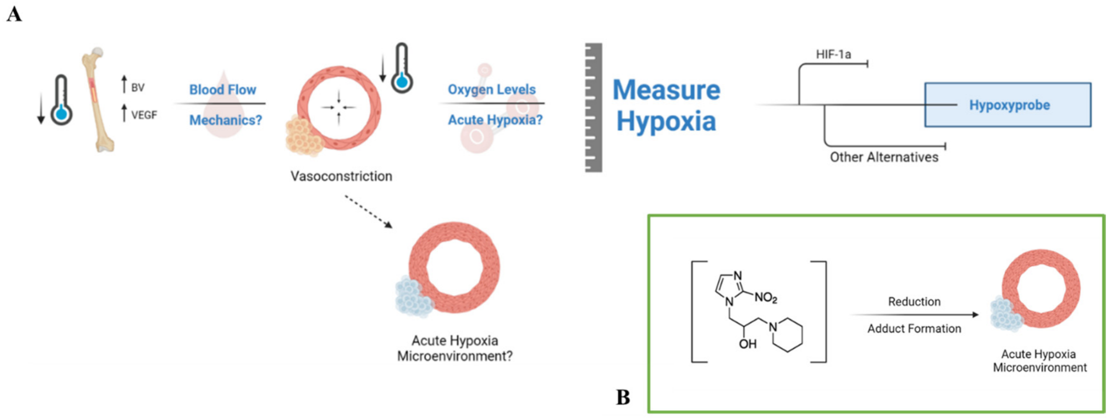

Figure S2. Progression in the rationale for pursuing hypoxia. **A)** Previous studies have shown that cold exposure leads to an increase in VEGF which affects vascularity. This guided us to consider blood flow mechanics and how a decrease in blood flow, contrary to common perception in that an increase in blood flow is preferred for bone healing, can facilitate bone repair by potentially inducing an acute hypoxic microenvironment from vasoconstriction. **B)** Pimonidazole tags hypoxic cells through an irreversible adduct formation that can then be detected via staining to provide relative hypoxic measurements of the impacts of Cold Therapy on the microenvironment of the cortical defect.

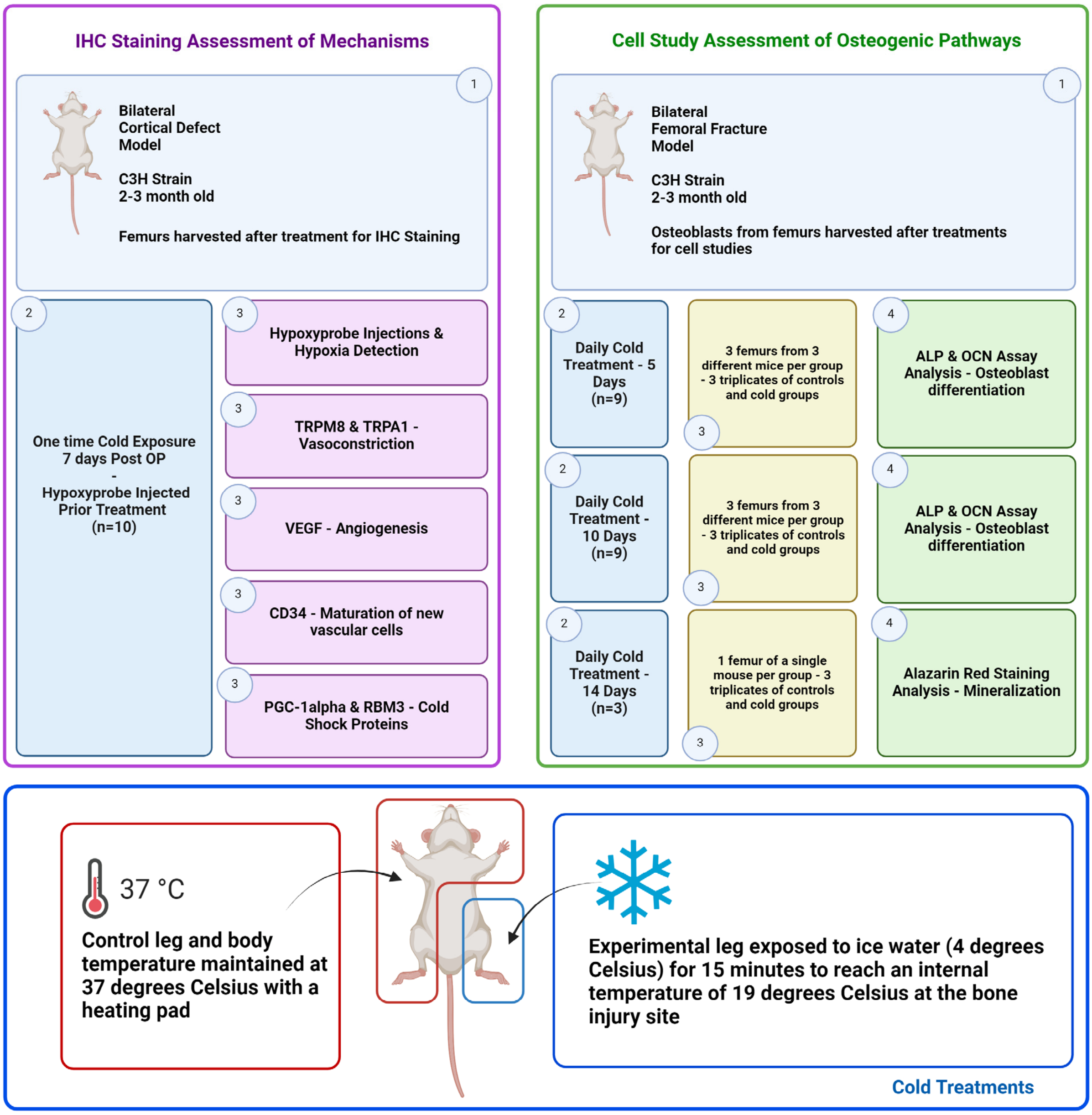

Figure S3. Schematic of the study design.

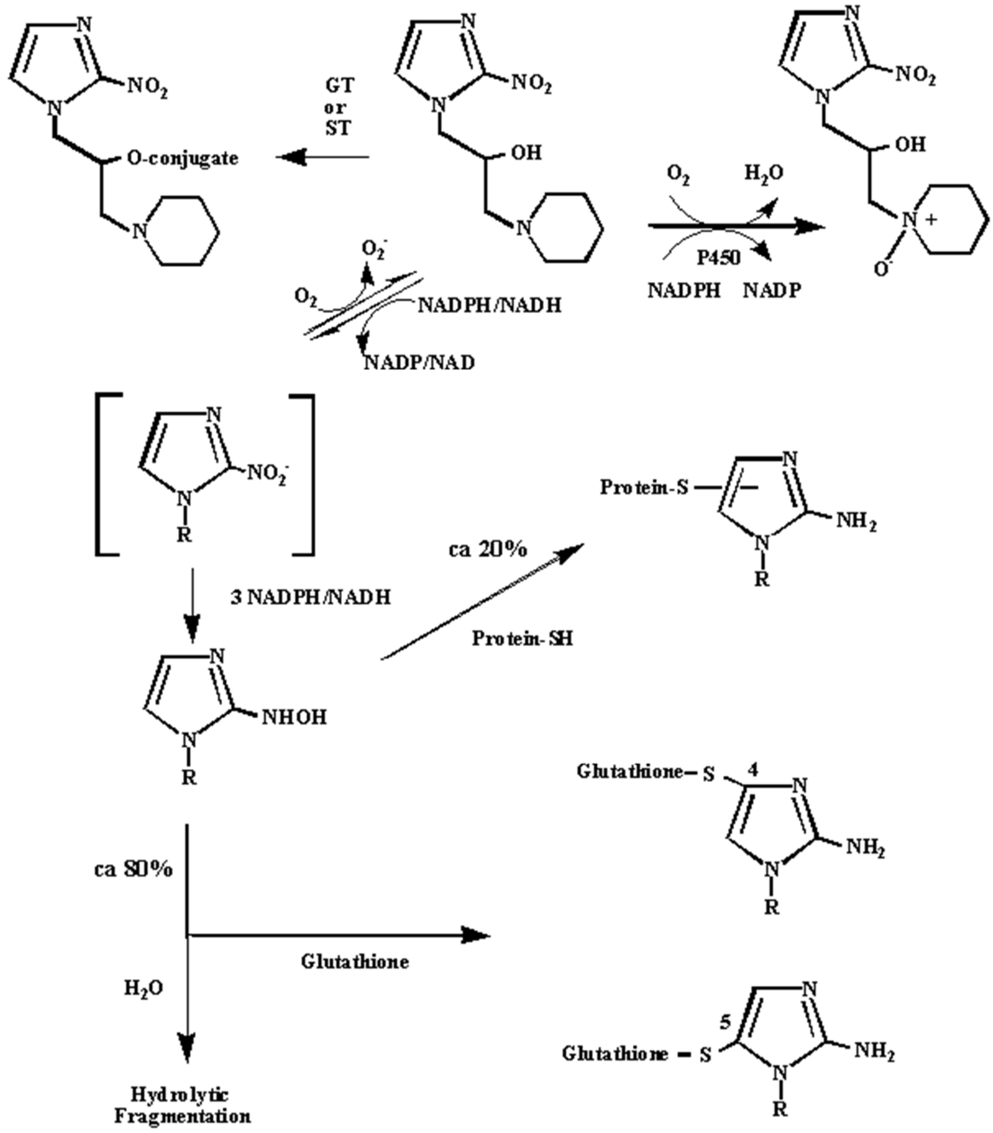

Figure S4. Hypoxyprobe Mechanism. Pimonidazole based detection of hypoxia relies on the formation of irreversible adducts in acute hypoxic environments.

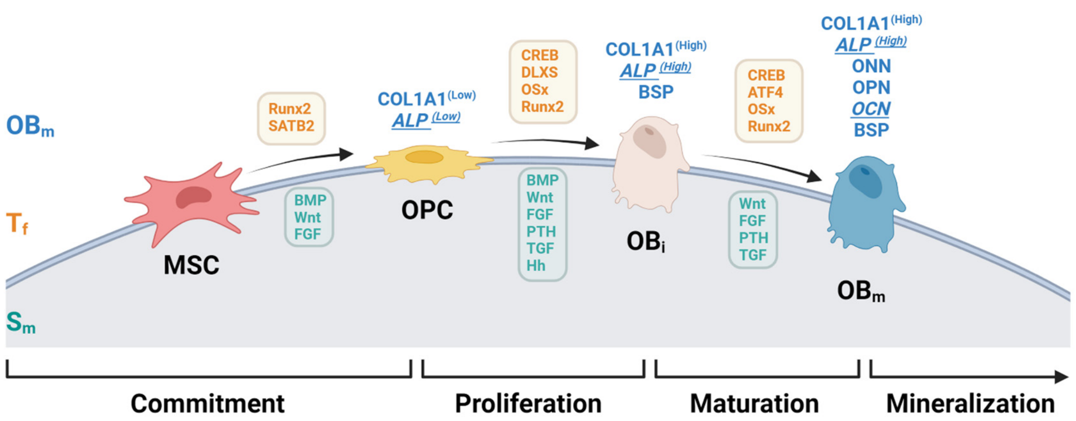

Figure S5. Differentiation of osteoblasts and biomarkers present.

Supplementary Information: Hypoxyprobe is a chemical compound designed to detect and measure tissue hypoxia, or low oxygen levels, in biological

samples. Pimonidazole hydrochloride, a 2-nitroimidazole derivative, serves as the active component due to its ability to undergo reduction in hypoxic conditions. Upon administration, typically through injection, pimonidazole diffuses throughout tissues. In normoxic conditions, it remains unaltered; however, under hypoxic conditions, it undergoes reduction reactions facilitated by cellular reductases. This reduced form becomes chemically reactive, binding to thiol groups on proteins, peptides, and amino acids within hypoxic cells, forming stable irreversible adducts in regions where oxygen levels are critically low [37]. These pimonidazole-protein adducts can then be detected using specific antibodies through immunohistochemical techniques, such as fluorescent or chromogenic staining, allowing for the visualization of hypoxic regions in tissue samples. The extent of hypoxia is quantified by measuring the intensity and distribution of the staining, which correlates with the level of pimonidazole binding and the severity and distribution of hypoxia within the tissue [37].

This pimonidazole-based staining approach is particularly effective in quantifying acute hypoxic induction due to the compound's properties. Acute hypoxia is associated with fluctuating partial oxygen pressure (pO2) resulting from blood flow instabilities [37]. Cells experiencing fluctuating hypoxia are typically located near blood vessels and at a relatively high, weakly basic pH. In these conditions, weakly basic 2-nitroimidazole hypoxic markers like pimonidazole are concentrated within these cells, leading to higher levels of binding compared to hypoxia markers lacking weakly basic moieties. As such, pimonidazole is highly effective for detecting acute hypoxia [64].
